# Crystal structure of *N*-[4-amino-5-cyano-6-(methyl­sulfan­yl)pyridin-2-yl]-2-(cyclo­hexyl­sulfan­yl)acetamide

**DOI:** 10.1107/S1600536814018534

**Published:** 2014-08-20

**Authors:** Joel T. Mague, Shaaban K. Mohamed, Mehmet Akkurt, Bahgat R. M. Hussein, Mustafa R. Albayati

**Affiliations:** a Department of Chemistry, Tulane University, New Orleans, LA 70118, USA; b Chemistry and Environmental Division, Manchester Metropolitan University, Manchester, M1 5GD, England; c Department of Physics, Faculty of Sciences, Erciyes University, 38039 Kayseri, Turkey; d Chemistry Department, Faculty of Science, Sohag University, 82524 Sohag, Egypt; e Kirkuk University, College of Science, Department of Chemistry, Kirkuk, Iraq

**Keywords:** crystal structure, acetamido, cyclo­hexyl­sulfan­yl, hydrogen bonds, π–π stacking

## Abstract

In the title mol­ecule, C_15_H_20_N_4_OS_2_, the acetamido fragment is nearly coplanar with the pyridyl ring [C—N—C—C torsion angle = −4.1 (2)°], while the cyclo­hexyl­sulfanyl portion protrudes from this plane [N—C—C—S torsion angle = −40.8 (6)°]. In the crystal, alternating pairwise N—H⋯O and N—H⋯N hydrogen bonds across inversion centres form chains along [101], which are associated into stepped layers *via* offset π–π stacking between pyridyl rings [centroid–centroid distance = 3.566 (1) Å]. The cyclo­hexyl group and the two atoms of the S—C bond attached to it are disordered over two sets of sites with site-occupancy factors of 0.8845 (18) and 0.1155 (18).

## Related literature   

For the diverse biological properties of pyridine-containing compounds see: Patrick & Kinsman (1996 ▶); Hishmat *et al.* (1990 ▶); Paronikyan *et al.* (2002 ▶); Bernardino *et al.* (2007 ▶); Doshi *et al.* (1999 ▶); Jemmezi *et al.* (2014 ▶); Mamolo *et al.* (2004 ▶); Bhatt *et al.* (2001 ▶). For the structure of a related compound, see: Akkurt *et al.* (2014 ▶).
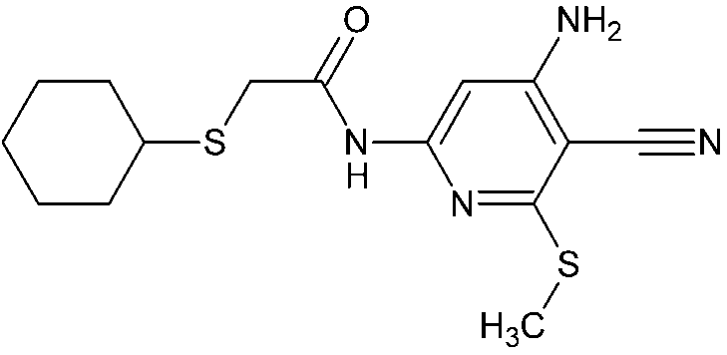


## Experimental   

### Crystal data  

C_15_H_20_N_4_OS_2_
*M* *_r_* = 336.47
Monoclinic, 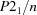

*a* = 7.2269 (8) Å
*b* = 24.655 (3) Å
*c* = 9.6933 (11) Å
β = 92.5330 (17)°
*V* = 1725.5 (3) Å^3^
*Z* = 4
Mo *K*α radiation
μ = 0.32 mm^−1^
*T* = 150 K
0.23 × 0.18 × 0.08 mm

### Data collection  

Bruker SMART APEX CCD diffractometer
Absorption correction: multi-scan (*SADABS*; Bruker, 2013 ▶) *T*_min_ = 0.93, *T*_max_ = 0.97
31462 measured reflections
4538 independent reflections
3713 reflections with *I* > 2σ(*I*)
*R*_int_ = 0.047

### Refinement  

*R*[*F* ^2^ > 2σ(*F* ^2^)] = 0.040
*wR*(*F* ^2^) = 0.106
*S* = 1.02
4538 reflections
225 parameters
68 restraints
H-atom parameters constrained
Δρ_max_ = 0.36 e Å^−3^
Δρ_min_ = −0.26 e Å^−3^

### 

Data collection: *APEX2* (Bruker, 2013 ▶); cell refinement: *SAINT* (Bruker, 2013 ▶); data reduction: *SAINT*; program(s) used to solve structure: *SHELXS2014* (Sheldrick, 2008 ▶); program(s) used to refine structure: *SHELXL2014* (Sheldrick, 2008 ▶); molecular graphics: *SHELXTL* (Sheldrick, 2008 ▶); software used to prepare material for publication: *SHELXTL*.

## Supplementary Material

Crystal structure: contains datablock(s) global, I. DOI: 10.1107/S1600536814018534/bh2502sup1.cif

Structure factors: contains datablock(s) I. DOI: 10.1107/S1600536814018534/bh2502Isup2.hkl

Supporting information file. DOI: 10.1107/S1600536814018534/bh2502Isup3.cml

**Fig. 1.**
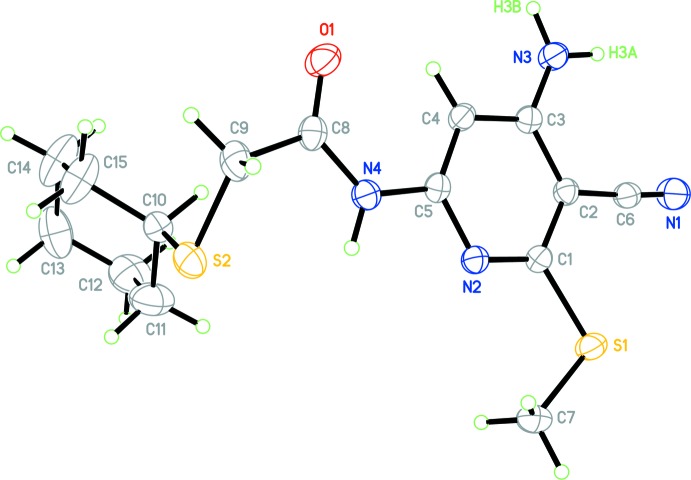
The title molecule showing displacement ellipsoids at the 50% probability level. Only the major component of the disorder is shown.

. DOI: 10.1107/S1600536814018534/bh2502fig1.tif
The title mol­ecule showing displacement ellipsoids at the 50% probability level. Only the major component of the disorder is shown.

**Fig. 2.**
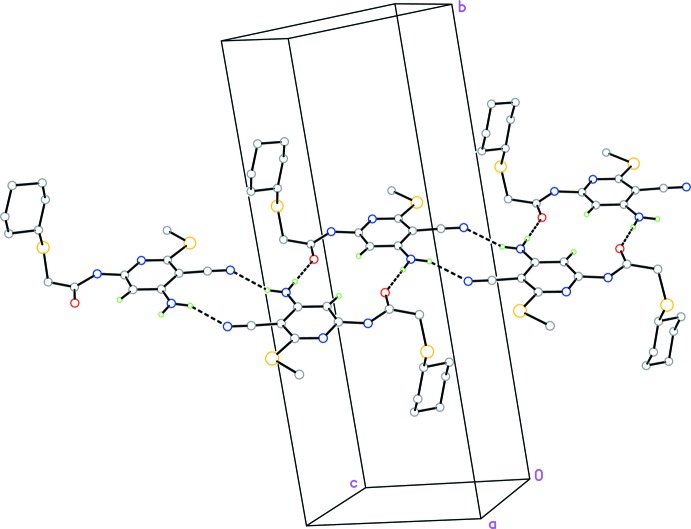
Perspective view of a portion of the hydrogen-bonded chain.

**Fig. 3.**
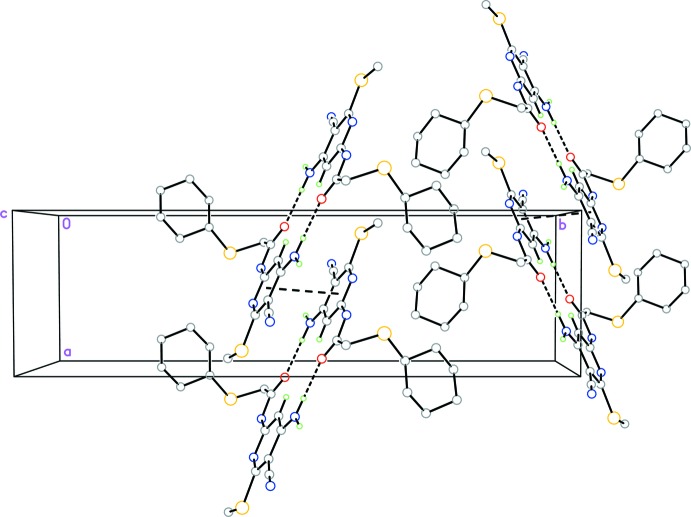
Packing viewed down the c axis, showing the pairwise N—H···O hydrogen bonding and the offset π-stacking interactions as dashed lines.

. DOI: 10.1107/S1600536814018534/bh2502fig2.tif
Perspective view of a portion of the hydrogen-bonded chain.

c . DOI: 10.1107/S1600536814018534/bh2502fig3.tif
Packing viewed down the *c* axis, showing the pairwise N—H⋯O hydrogen bonding and the offset π-stacking inter­actions as dashed lines.

CCDC reference: 1019463

Additional supporting information:  crystallographic information; 3D view; checkCIF report

## Figures and Tables

**Table 1:** Hydrogen-bond geometry (Å, °)

*D*—H⋯*A*	*D*—H	H⋯*A*	*D*⋯*A*	*D*—H⋯*A*
N3—H3*B*⋯O1^i^	0.91	1.97	2.8792 (17)	179
N3—H3*A*⋯N1^ii^	0.91	2.22	3.0640 (19)	155

Symmetry codes: (i) 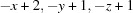; (ii) 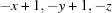.

## supplementary crystallographic information

### S1. Comment

A large number of heterocyclic compounds containing pyridine rings are associated with diverse pharmacological properties such as antifungal (Patrick & Kinsman, 1996; Hishmat *et al.*, 1990), anticancer, anticonvulsant (Paronikyan *et al.*, 2002), antiviral (Bernardino *et al.*, 2007), antibacterial, antimicrobial (Doshi *et al.*, 1999; Jemmezi *et al.*, 2014), antimycobacterial (Mamolo *et al.*, 2004) and insecticidal activities (Bhatt *et al.*, 2001). In this connection, and as part of our on-going study on synthesis of bio-active heterocyclic molecules, we herein report the synthesis and crystal determination of the title compound.

In the title molecule (Fig. 1), the acetamido fragment is nearly coplanar with the pyridyl ring [C8—N4—C5—C4 torsion angle = -4.1 (2)°], possibly aided by a weak C4—H4···O1 interaction (Table 1), while the cyclohexylsulfanyl portion protrudes from this plane [N4—C8—C9—S2 torsion angle = -40.8 (6)°]. The main disordered part of the cyclohexyl group adopts the chair conformation with puckering parameters Q = 0.578 (3) Å, θ = 177.6 (3)°, and φ = 311 (3)°. The minor disordered part of the cyclohexyl group exhibits a distorted chair conformation with puckering parameters Q = 0.60 (2) Å, θ = 11.3 (19) °, and φ = 173 (10) °. All the bond lengths and bond angles are normal and comparable with those reported for a related compound (Akkurt *et al.*, 2014).

Alternating, pairwise N3—H3B···O1 and N3—H3A···N1 hydrogen bonds across centres of symmetry form chains (Fig. 2 and Table 1) which are associated into stepped layers *via* offset π-stacking between pyridyl rings [Fig. 3; interplanar distance = 3.384 (1) Å; *Cg*···*Cg^i^* = 3.566 (1) Å, where *Cg* is the centroid of the C1···C5/N2 ring; *i*: 1 - *x*, 1 - *y*, 1 - *z*]. Adjacent stacks are inclined to one another by approximately 62°.

### S2. Experimental

A mixture of 1 mmol (257 mg) of *N*-[4-amino-5-cyano-6-(methylthio)pyridin-2-yl]-2-chloroacetamide and 1 mmol (116 mg) of cyclohexanethiol in 30 ml e thanol along with few drops of triethylamine (TEA) as a catalyst was refluxed for 3 h at 350 K. The reaction mixture was allowed to cool down at room temperature and the excess solvent was evaporated under reduced pressure. The resulting solid was filtered off, dried and recrystallized from benzene, to afford colourless crystals (92% yield) suitable for X-ray diffraction. *M*.p. 463 – 465 K.

IR (ν_max_, cm^-1^): 3470, 3325, 3215, (NH_2_+NH), 2928 (CH *_aliph_*_._), 2210 (C≡N), 1689 (C=O*_amidic_*); ^1^H-NMR (DMSO-*d_6_*), δ, p.p.m.: 10.27 (s, 1H, NH; exchanged by D_2_O), 7.30 (s, 1H, CH *_pyridyl_*), 6.99 (s, 2H, NH_2_; exchanged by D_2_O), 3.41 (s, 2H, COCH_2_), 2.84–2.82 (m, 1H, CH *_cyclohexyl_*), 2.52 (s, 3H, SCH_3_), 1.95–1.93 (m, 2H, CH_2_*_cyclohexyl_*), 1.69 (m, 2H, CH_2_*_cyclohexyl_*), 1.56–1.54 (m, 1H, CH *_cyclohexyl_*), 1.28–1.24 (m, 5H, 2CH_2_+CH *_cyclohexyl_*).

### S3. Refinement

H atoms attached to carbon were placed in calculated positions (C—H = 0.95 - 0.99 Å) while those attached to nitrogen were placed in locations derived from a difference map and their parameters adjusted to give N—H = 0.91 Å. All H atoms were included as riding contributions with isotropic displacement parameters 1.2 - 1.5 times those of the attached atoms. The cyclohexyl group, S2 and C9 are disordered over two sites [site-occupancy factors are 0.8845 (18) and 0.1155 (18)]. The components of the disorder were refined with restraints that their geometries be the same (Sheldrick, 2008)

### Crystal data

**Table:**

C_15_H_20_N_4_OS_2_	*D*_x_ = 1.295 Mg m^−^^3^
*M**_r_* = 336.47	Melting point: 463 K
Monoclinic, *P*2_1_/*n*	Mo *K*α radiation, λ = 0.71073 Å
*a* = 7.2269 (8) Å	Cell parameters from 9975 reflections
*b* = 24.655 (3) Å	θ = 2.3–29.0°
*c* = 9.6933 (11) Å	µ = 0.32 mm^−^^1^
β = 92.5330 (17)°	*T* = 150 K
*V* = 1725.5 (3) Å^3^	Plate, colourless
*Z* = 4	0.23 × 0.18 × 0.08 mm
*F*(000) = 712	

### Data collection

**Table:**

Bruker SMART APEX CCD diffractometer	4538 independent reflections
Radiation source: fine-focus sealed tube	3713 reflections with *I* > 2σ(*I*)
Graphite monochromator	*R*_int_ = 0.047
Detector resolution: 8.3660 pixels mm^-1^	θ_max_ = 29.0°, θ_min_ = 2.3°
φ and ω scans	*h* = −9→9
Absorption correction: multi-scan (*SADABS*; Bruker, 2013)	*k* = −33→33
*T*_min_ = 0.93, *T*_max_ = 0.97	*l* = −13→13
31462 measured reflections	

### Refinement

**Table:**

Refinement on *F*^2^	Primary atom site location: structure-invariant direct methods
Least-squares matrix: full	Secondary atom site location: difference Fourier map
*R*[*F*^2^ > 2σ(*F*^2^)] = 0.040	Hydrogen site location: mixed
*wR*(*F*^2^) = 0.106	H-atom parameters constrained
*S* = 1.02	*w* = 1/[σ^2^(*F*_o_^2^) + (0.0492*P*)^2^ + 0.7171*P*] where *P* = (*F*_o_^2^ + 2*F*_c_^2^)/3
4538 reflections	(Δ/σ)_max_ = 0.006
225 parameters	Δρ_max_ = 0.36 e Å^−^^3^
68 restraints	Δρ_min_ = −0.26 e Å^−^^3^
0 constraints	

### Special details

**Table:**

Experimental. The diffraction data were obtained from 3 sets of 400 frames, each of width 0.5° in ω, collected at φ = 0.00, 90.00 and 180.00° and 2 sets of 800 frames, each of width 0.45° in φ, collected at ω = -30.00 and 210.00°. The scan time was 40 sec/frame.

### Fractional atomic coordinates and isotropic or equivalent isotropic displacement parameters (Å^2^)

**Table:**

	*x*	*y*	*z*	*U*_iso_*/*U*_eq_	Occ. (<1)
S1	0.12721 (5)	0.61034 (2)	0.34743 (4)	0.03110 (11)	
O1	0.91811 (16)	0.53918 (5)	0.71493 (12)	0.0356 (3)	
N1	0.3292 (2)	0.54925 (6)	0.04030 (14)	0.0378 (3)	
N2	0.41788 (16)	0.59429 (5)	0.52327 (12)	0.0235 (2)	
N3	0.72278 (17)	0.50958 (5)	0.23714 (13)	0.0287 (3)	
H3A	0.6773	0.4994	0.1520	0.034*	
H3B	0.8363	0.4940	0.2514	0.034*	
N4	0.64424 (17)	0.58430 (5)	0.69315 (12)	0.0264 (3)	
H4A	0.5680	0.6065	0.7397	0.032*	
C1	0.35074 (19)	0.58641 (5)	0.39522 (15)	0.0227 (3)	
C2	0.44875 (19)	0.55937 (5)	0.29318 (14)	0.0218 (3)	
C3	0.62544 (18)	0.53707 (5)	0.32894 (14)	0.0222 (3)	
C4	0.69559 (19)	0.54512 (6)	0.46531 (14)	0.0237 (3)	
H4	0.8137	0.5314	0.4948	0.028*	
C5	0.58845 (19)	0.57337 (6)	0.55479 (14)	0.0226 (3)	
C6	0.37713 (19)	0.55438 (6)	0.15424 (15)	0.0258 (3)	
C7	0.0634 (2)	0.64195 (7)	0.50548 (17)	0.0332 (3)	
H7A	0.0792	0.6160	0.5817	0.050*	
H7B	−0.0664	0.6535	0.4968	0.050*	
H7C	0.1425	0.6736	0.5242	0.050*	
C8	0.8003 (2)	0.56755 (6)	0.76461 (15)	0.0258 (3)	
S2	0.74307 (10)	0.65352 (3)	0.94799 (6)	0.03506 (15)	0.8845 (18)
C9	0.8162 (10)	0.5850 (2)	0.9147 (11)	0.0327 (9)	0.8845 (18)
H9A	0.9470	0.5811	0.9481	0.039*	0.8845 (18)
H9B	0.7413	0.5600	0.9693	0.039*	0.8845 (18)
C10	0.9173 (2)	0.69109 (7)	0.85620 (18)	0.0303 (4)	0.8845 (18)
H10	0.9378	0.6717	0.7675	0.036*	0.8845 (18)
C11	0.8422 (3)	0.74748 (9)	0.8213 (3)	0.0504 (6)	0.8845 (18)
H11A	0.8143	0.7667	0.9075	0.061*	0.8845 (18)
H11B	0.7255	0.7441	0.7645	0.061*	0.8845 (18)
C12	0.9827 (4)	0.78041 (10)	0.7421 (3)	0.0535 (6)	0.8845 (18)
H12A	1.0040	0.7625	0.6527	0.064*	0.8845 (18)
H12B	0.9323	0.8171	0.7225	0.064*	0.8845 (18)
C13	1.1633 (4)	0.78515 (11)	0.8244 (3)	0.0600 (7)	0.8845 (18)
H13A	1.1437	0.8053	0.9110	0.072*	0.8845 (18)
H13B	1.2532	0.8057	0.7707	0.072*	0.8845 (18)
C14	1.2412 (3)	0.72892 (12)	0.8587 (3)	0.0629 (7)	0.8845 (18)
H14A	1.3574	0.7327	0.9159	0.075*	0.8845 (18)
H14B	1.2708	0.7102	0.7721	0.075*	0.8845 (18)
C15	1.1027 (3)	0.69463 (11)	0.9368 (3)	0.0555 (6)	0.8845 (18)
H15A	1.1534	0.6577	0.9517	0.067*	0.8845 (18)
H15B	1.0842	0.7111	1.0284	0.067*	0.8845 (18)
S2A	0.6907 (8)	0.6447 (2)	0.9744 (5)	0.03506 (15)	0.1155 (18)
C9A	0.833 (9)	0.589 (2)	0.918 (9)	0.0327 (9)	0.1155 (18)
H9A1	0.9638	0.6007	0.9302	0.039*	0.1155 (18)
H9A2	0.8151	0.5584	0.9817	0.039*	0.1155 (18)
C10A	0.8290 (17)	0.7034 (5)	0.9292 (12)	0.0303 (4)	0.1155 (18)
H10A	0.7526	0.7352	0.9560	0.036*	0.1155 (18)
C11A	0.854 (3)	0.7113 (6)	0.7781 (15)	0.0504 (6)	0.1155 (18)
H11C	0.7317	0.7120	0.7283	0.061*	0.1155 (18)
H11D	0.9259	0.6806	0.7420	0.061*	0.1155 (18)
C12A	0.955 (3)	0.7639 (8)	0.753 (3)	0.0535 (6)	0.1155 (18)
H12C	0.8818	0.7945	0.7879	0.064*	0.1155 (18)
H12D	0.9664	0.7690	0.6524	0.064*	0.1155 (18)
C13A	1.148 (3)	0.7645 (10)	0.8242 (19)	0.0600 (7)	0.1155 (18)
H13C	1.2163	0.7980	0.8013	0.072*	0.1155 (18)
H13D	1.2214	0.7326	0.7971	0.072*	0.1155 (18)
C14A	1.109 (2)	0.7627 (8)	0.9781 (18)	0.0629 (7)	0.1155 (18)
H14C	1.2265	0.7651	1.0337	0.075*	0.1155 (18)
H14D	1.0308	0.7941	1.0018	0.075*	0.1155 (18)
C15A	1.009 (2)	0.7104 (8)	1.013 (2)	0.0555 (6)	0.1155 (18)
H15C	1.0912	0.6792	0.9947	0.067*	0.1155 (18)
H15D	0.9836	0.7102	1.1120	0.067*	0.1155 (18)

### Atomic displacement parameters (Å^2^)

**Table:**

	*U*^11^	*U*^22^	*U*^33^	*U*^12^	*U*^13^	*U*^23^
S1	0.02353 (18)	0.0410 (2)	0.0283 (2)	0.00880 (15)	−0.00329 (14)	−0.00609 (15)
O1	0.0297 (6)	0.0485 (7)	0.0281 (6)	0.0102 (5)	−0.0041 (4)	−0.0026 (5)
N1	0.0409 (8)	0.0456 (8)	0.0261 (7)	0.0097 (6)	−0.0061 (6)	−0.0053 (6)
N2	0.0222 (6)	0.0265 (6)	0.0217 (6)	0.0017 (4)	−0.0002 (4)	−0.0023 (5)
N3	0.0253 (6)	0.0402 (7)	0.0206 (6)	0.0068 (5)	−0.0003 (5)	−0.0054 (5)
N4	0.0270 (6)	0.0320 (6)	0.0201 (6)	0.0037 (5)	−0.0010 (5)	−0.0048 (5)
C1	0.0201 (6)	0.0235 (6)	0.0245 (7)	−0.0005 (5)	0.0002 (5)	−0.0004 (5)
C2	0.0222 (6)	0.0233 (6)	0.0199 (6)	0.0000 (5)	−0.0004 (5)	−0.0003 (5)
C3	0.0216 (6)	0.0239 (6)	0.0212 (6)	−0.0013 (5)	0.0023 (5)	0.0002 (5)
C4	0.0195 (6)	0.0288 (7)	0.0226 (7)	0.0012 (5)	−0.0007 (5)	−0.0013 (5)
C5	0.0225 (6)	0.0248 (6)	0.0204 (6)	−0.0018 (5)	−0.0012 (5)	−0.0007 (5)
C6	0.0242 (7)	0.0277 (7)	0.0253 (7)	0.0043 (5)	−0.0006 (5)	−0.0019 (5)
C7	0.0285 (7)	0.0363 (8)	0.0353 (8)	0.0062 (6)	0.0058 (6)	−0.0048 (7)
C8	0.0280 (7)	0.0270 (7)	0.0221 (7)	−0.0040 (5)	−0.0021 (5)	0.0018 (5)
S2	0.0358 (3)	0.0437 (3)	0.0258 (3)	0.0014 (2)	0.0031 (2)	−0.0104 (2)
C9	0.0430 (19)	0.0336 (14)	0.0210 (10)	−0.0013 (13)	−0.0052 (14)	0.0001 (11)
C10	0.0315 (9)	0.0308 (8)	0.0283 (9)	0.0031 (7)	−0.0019 (7)	−0.0059 (7)
C11	0.0486 (12)	0.0362 (10)	0.0669 (15)	0.0101 (9)	0.0078 (11)	−0.0019 (10)
C12	0.0639 (16)	0.0350 (14)	0.0615 (15)	0.0021 (12)	0.0024 (12)	0.0057 (13)
C13	0.0766 (17)	0.0525 (16)	0.0504 (13)	−0.0250 (15)	−0.0012 (12)	−0.0093 (13)
C14	0.0373 (12)	0.0873 (19)	0.0622 (16)	−0.0158 (12)	−0.0173 (11)	0.0164 (14)
C15	0.0405 (12)	0.0721 (16)	0.0521 (14)	−0.0090 (11)	−0.0170 (10)	0.0152 (12)
S2A	0.0358 (3)	0.0437 (3)	0.0258 (3)	0.0014 (2)	0.0031 (2)	−0.0104 (2)
C9A	0.0430 (19)	0.0336 (14)	0.0210 (10)	−0.0013 (13)	−0.0052 (14)	0.0001 (11)
C10A	0.0315 (9)	0.0308 (8)	0.0283 (9)	0.0031 (7)	−0.0019 (7)	−0.0059 (7)
C11A	0.0486 (12)	0.0362 (10)	0.0669 (15)	0.0101 (9)	0.0078 (11)	−0.0019 (10)
C12A	0.0639 (16)	0.0350 (14)	0.0615 (15)	0.0021 (12)	0.0024 (12)	0.0057 (13)
C13A	0.0766 (17)	0.0525 (16)	0.0504 (13)	−0.0250 (15)	−0.0012 (12)	−0.0093 (13)
C14A	0.0373 (12)	0.0873 (19)	0.0622 (16)	−0.0158 (12)	−0.0173 (11)	0.0164 (14)
C15A	0.0405 (12)	0.0721 (16)	0.0521 (14)	−0.0090 (11)	−0.0170 (10)	0.0152 (12)

### Geometric parameters (Å, º)

**Table:**

S1—C1	1.7624 (14)	C12—C13	1.505 (4)
S1—C7	1.7972 (16)	C12—H12A	0.9900
O1—C8	1.2173 (18)	C12—H12B	0.9900
N1—C6	1.150 (2)	C13—C14	1.527 (4)
N2—C1	1.3269 (18)	C13—H13A	0.9900
N2—C5	1.3586 (18)	C13—H13B	0.9900
N3—C3	1.3422 (18)	C14—C15	1.535 (3)
N3—H3A	0.9101	C14—H14A	0.9900
N3—H3B	0.9104	C14—H14B	0.9900
N4—C8	1.3616 (19)	C15—H15A	0.9900
N4—C5	1.4093 (18)	C15—H15B	0.9900
N4—H4A	0.9098	S2A—C9A	1.807 (18)
C1—C2	1.4094 (19)	S2A—C10A	1.823 (12)
C2—C3	1.4190 (19)	C9A—H9A1	0.9900
C2—C6	1.427 (2)	C9A—H9A2	0.9900
C3—C4	1.4087 (19)	C10A—C11A	1.496 (13)
C4—C5	1.3770 (19)	C10A—C15A	1.511 (12)
C4—H4	0.9500	C10A—H10A	1.0000
C7—H7A	0.9800	C11A—C12A	1.512 (13)
C7—H7B	0.9800	C11A—H11C	0.9900
C7—H7C	0.9800	C11A—H11D	0.9900
C8—C9	1.516 (10)	C12A—C13A	1.529 (14)
C8—C9A	1.59 (8)	C12A—H12C	0.9900
S2—C9	1.803 (4)	C12A—H12D	0.9900
S2—C10	1.826 (2)	C13A—C14A	1.533 (14)
C9—H9A	0.9900	C13A—H13C	0.9900
C9—H9B	0.9900	C13A—H13D	0.9900
C10—C15	1.523 (3)	C14A—C15A	1.520 (13)
C10—C11	1.525 (3)	C14A—H14C	0.9900
C10—H10	1.0000	C14A—H14D	0.9900
C11—C12	1.532 (4)	C15A—H15C	0.9900
C11—H11A	0.9900	C15A—H15D	0.9900
C11—H11B	0.9900		
			
C1—S1—C7	100.83 (7)	C12—C13—H13A	109.6
C1—N2—C5	116.45 (12)	C14—C13—H13A	109.6
C3—N3—H3A	124.2	C12—C13—H13B	109.6
C3—N3—H3B	127.4	C14—C13—H13B	109.6
H3A—N3—H3B	107.9	H13A—C13—H13B	108.1
C8—N4—C5	128.45 (12)	C13—C14—C15	111.4 (2)
C8—N4—H4A	115.9	C13—C14—H14A	109.3
C5—N4—H4A	115.6	C15—C14—H14A	109.3
N2—C1—C2	123.46 (13)	C13—C14—H14B	109.3
N2—C1—S1	119.33 (10)	C15—C14—H14B	109.3
C2—C1—S1	117.21 (11)	H14A—C14—H14B	108.0
C1—C2—C3	119.11 (12)	C10—C15—C14	110.86 (18)
C1—C2—C6	122.05 (12)	C10—C15—H15A	109.5
C3—C2—C6	118.83 (12)	C14—C15—H15A	109.5
N3—C3—C4	121.05 (13)	C10—C15—H15B	109.5
N3—C3—C2	121.74 (13)	C14—C15—H15B	109.5
C4—C3—C2	117.21 (12)	H15A—C15—H15B	108.1
C5—C4—C3	118.24 (13)	C9A—S2A—C10A	102 (3)
C5—C4—H4	120.9	C8—C9A—S2A	118 (4)
C3—C4—H4	120.9	C8—C9A—H9A1	107.7
N2—C5—C4	125.47 (13)	S2A—C9A—H9A1	107.7
N2—C5—N4	111.10 (12)	C8—C9A—H9A2	107.7
C4—C5—N4	123.44 (13)	S2A—C9A—H9A2	107.7
N1—C6—C2	176.06 (16)	H9A1—C9A—H9A2	107.1
S1—C7—H7A	109.5	C11A—C10A—C15A	111.7 (13)
S1—C7—H7B	109.5	C11A—C10A—S2A	115.5 (9)
H7A—C7—H7B	109.5	C15A—C10A—S2A	115.5 (9)
S1—C7—H7C	109.5	C11A—C10A—H10A	104.2
H7A—C7—H7C	109.5	C15A—C10A—H10A	104.2
H7B—C7—H7C	109.5	S2A—C10A—H10A	104.2
O1—C8—N4	123.44 (14)	C10A—C11A—C12A	110.5 (17)
O1—C8—C9	121.2 (3)	C10A—C11A—H11C	109.6
N4—C8—C9	115.3 (3)	C12A—C11A—H11C	109.6
O1—C8—C9A	119.2 (18)	C10A—C11A—H11D	109.6
N4—C8—C9A	117.3 (17)	C12A—C11A—H11D	109.6
C9—S2—C10	100.0 (3)	H11C—C11A—H11D	108.1
C8—C9—S2	115.3 (6)	C11A—C12A—C13A	111.9 (17)
C8—C9—H9A	108.4	C11A—C12A—H12C	109.2
S2—C9—H9A	108.4	C13A—C12A—H12C	109.2
C8—C9—H9B	108.4	C11A—C12A—H12D	109.2
S2—C9—H9B	108.4	C13A—C12A—H12D	109.2
H9A—C9—H9B	107.5	H12C—C12A—H12D	107.9
C15—C10—C11	110.98 (18)	C12A—C13A—C14A	103 (2)
C15—C10—S2	112.88 (14)	C12A—C13A—H13C	111.1
C11—C10—S2	108.92 (14)	C14A—C13A—H13C	111.1
C15—C10—H10	108.0	C12A—C13A—H13D	111.1
C11—C10—H10	108.0	C14A—C13A—H13D	111.1
S2—C10—H10	108.0	H13C—C13A—H13D	109.0
C10—C11—C12	110.92 (19)	C15A—C14A—C13A	110.5 (17)
C10—C11—H11A	109.5	C15A—C14A—H14C	109.6
C12—C11—H11A	109.5	C13A—C14A—H14C	109.6
C10—C11—H11B	109.5	C15A—C14A—H14D	109.6
C12—C11—H11B	109.5	C13A—C14A—H14D	109.6
H11A—C11—H11B	108.0	H14C—C14A—H14D	108.1
C13—C12—C11	110.7 (2)	C10A—C15A—C14A	112.5 (13)
C13—C12—H12A	109.5	C10A—C15A—H15C	109.1
C11—C12—H12A	109.5	C14A—C15A—H15C	109.1
C13—C12—H12B	109.5	C10A—C15A—H15D	109.1
C11—C12—H12B	109.5	C14A—C15A—H15D	109.1
H12A—C12—H12B	108.1	H15C—C15A—H15D	107.8
C12—C13—C14	110.3 (2)		
			
C5—N2—C1—C2	−1.8 (2)	C10—S2—C9—C8	−65.2 (5)
C5—N2—C1—S1	177.97 (10)	C9—S2—C10—C15	−78.6 (4)
C7—S1—C1—N2	1.40 (13)	C9—S2—C10—C11	157.7 (4)
C7—S1—C1—C2	−178.84 (11)	C15—C10—C11—C12	56.0 (3)
N2—C1—C2—C3	2.8 (2)	S2—C10—C11—C12	−179.18 (18)
S1—C1—C2—C3	−176.92 (10)	C10—C11—C12—C13	−57.8 (3)
N2—C1—C2—C6	−176.20 (13)	C11—C12—C13—C14	57.8 (3)
S1—C1—C2—C6	4.05 (18)	C12—C13—C14—C15	−56.8 (3)
C1—C2—C3—N3	178.10 (13)	C11—C10—C15—C14	−54.6 (3)
C6—C2—C3—N3	−2.8 (2)	S2—C10—C15—C14	−177.19 (19)
C1—C2—C3—C4	−2.14 (19)	C13—C14—C15—C10	55.2 (3)
C6—C2—C3—C4	176.93 (13)	O1—C8—C9A—S2A	164 (3)
N3—C3—C4—C5	−179.59 (13)	N4—C8—C9A—S2A	−13 (6)
C2—C3—C4—C5	0.6 (2)	C10A—S2A—C9A—C8	−90 (5)
C1—N2—C5—C4	0.1 (2)	C9A—S2A—C10A—C11A	65 (3)
C1—N2—C5—N4	−179.93 (12)	C9A—S2A—C10A—C15A	−68 (3)
C3—C4—C5—N2	0.4 (2)	C15A—C10A—C11A—C12A	−51 (2)
C3—C4—C5—N4	−179.53 (13)	S2A—C10A—C11A—C12A	174.6 (14)
C8—N4—C5—N2	176.01 (14)	C10A—C11A—C12A—C13A	61 (3)
C8—N4—C5—C4	−4.1 (2)	C11A—C12A—C13A—C14A	−65 (3)
C5—N4—C8—O1	−0.2 (2)	C12A—C13A—C14A—C15A	62 (2)
C5—N4—C8—C9	−178.1 (4)	C11A—C10A—C15A—C14A	51 (2)
C5—N4—C8—C9A	176 (3)	S2A—C10A—C15A—C14A	−174.9 (14)
O1—C8—C9—S2	141.3 (3)	C13A—C14A—C15A—C10A	−58 (2)
N4—C8—C9—S2	−40.8 (6)		

### Hydrogen-bond geometry (Å, º)

**Table:**

*D*—H···*A*	*D*—H	H···*A*	*D*···*A*	*D*—H···*A*
N3—H3*B*···O1^i^	0.91	1.97	2.8792 (17)	179
N3—H3*A*···N1^ii^	0.91	2.22	3.0640 (19)	155
C4—H4···O1	0.95	2.24	2.8493 (18)	121
C7—H7*A*···O1^iii^	0.98	2.60	3.440 (2)	144

Symmetry codes: (i) −*x*+2, −*y*+1, −*z*+1; (ii) −*x*+1, −*y*+1, −*z*; (iii) *x*−1, *y*, *z*.
