# Supplementary figures and images for: Crystal structure of N-[4-amino-5-cyano-6-(methyl­sulfan­yl)pyridin-2-yl]-2-(cyclo­hexyl­sulfan­yl)acetamide
Source: Acta Crystallogr Sect E Struct Rep Online. 2014 Aug 20;70(Pt 9):o1031–2. doi: 10.1107/S1600536814018534 (PMC4186101; doi:10.1107/S1600536814018534)

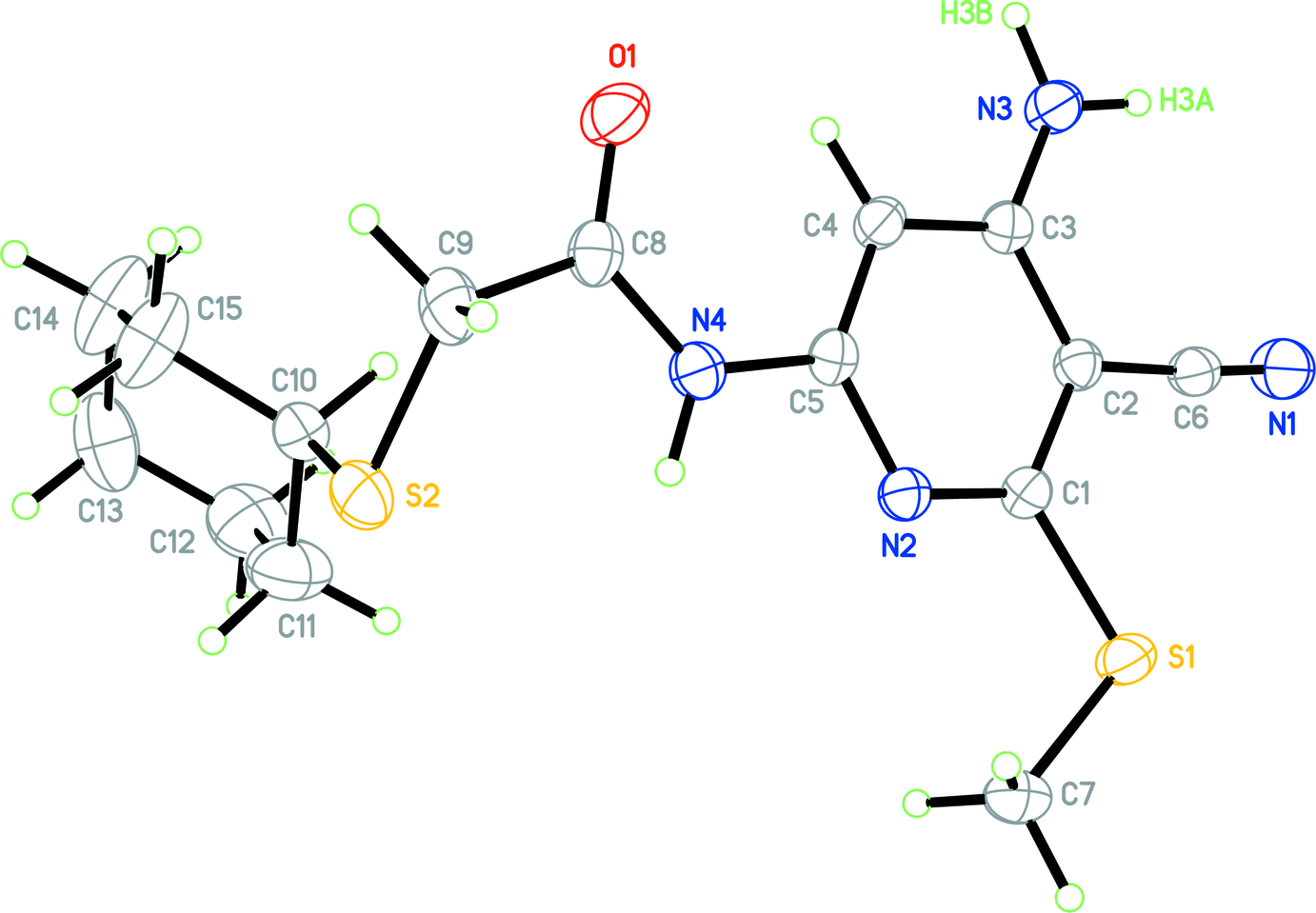

Supplement: Supplementary file 4 [file e-70-o1031-fig1.tif]

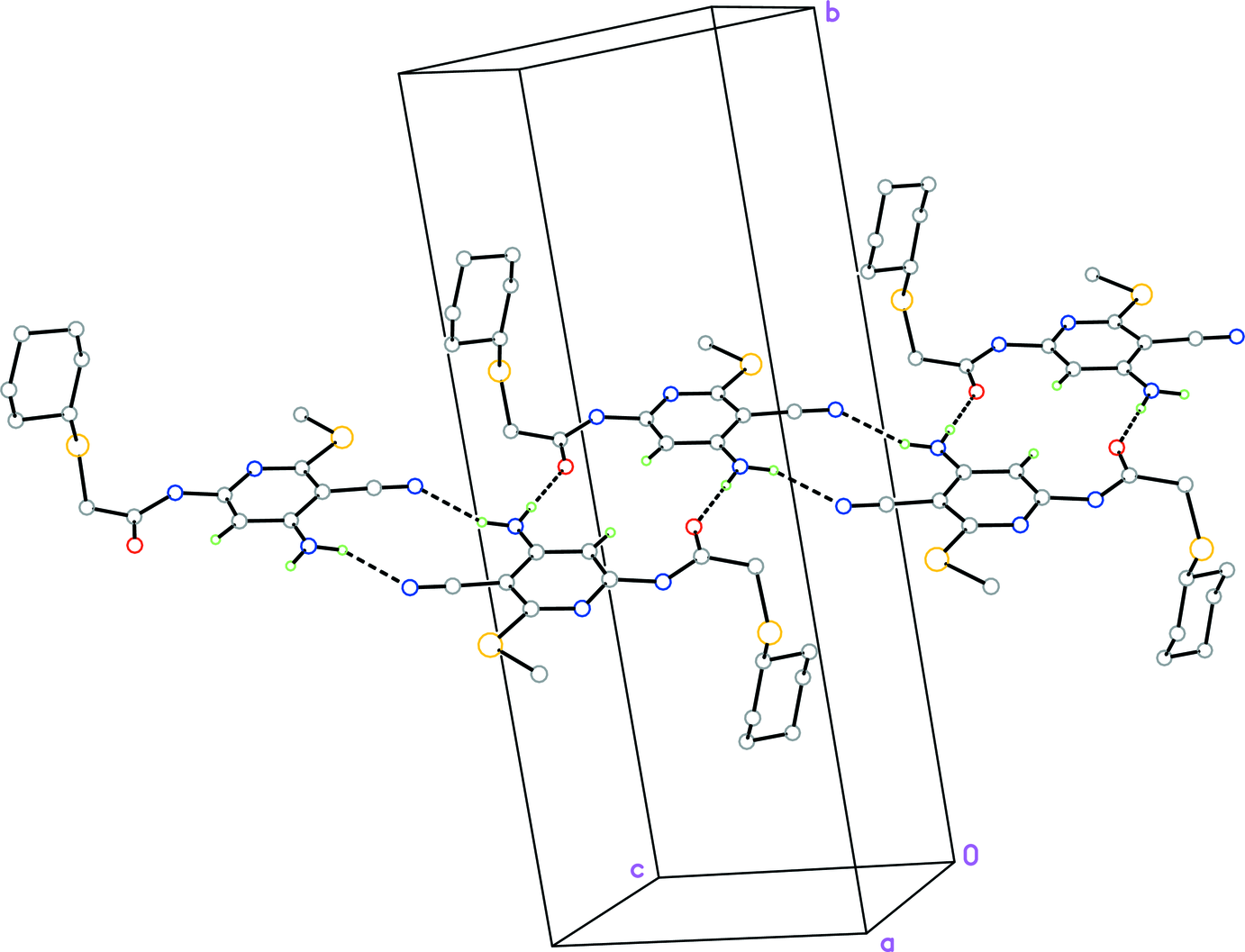

Supplement: Supplementary file 5 [file e-70-o1031-fig2.tif]

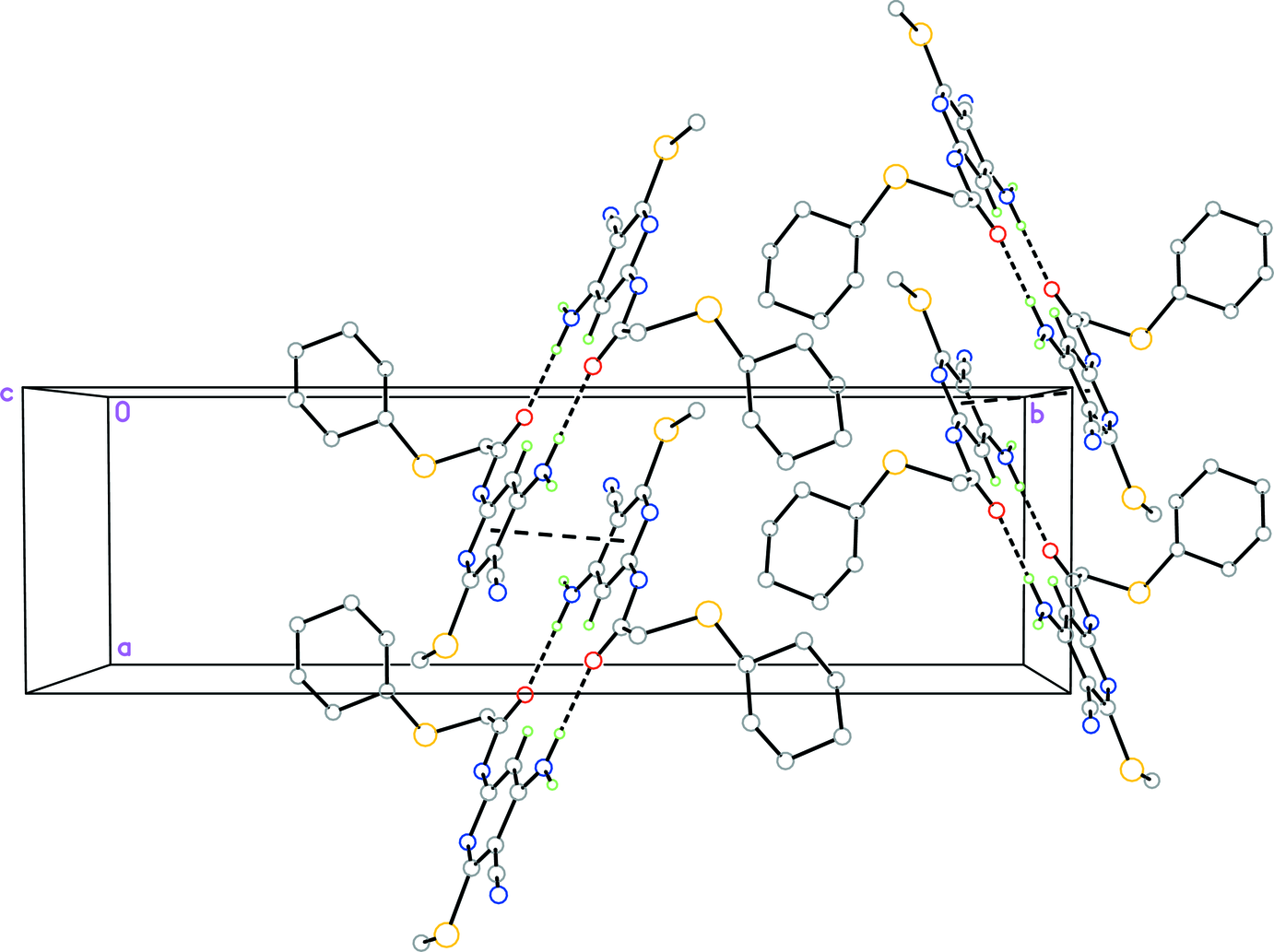

Supplement: Supplementary file 6 [file e-70-o1031-fig3.tif]
